# Finite Element Analysis of Interface Dependence on Nanomechanical Sensing

**DOI:** 10.3390/s20051518

**Published:** 2020-03-10

**Authors:** Kosuke Minami, Genki Yoshikawa

**Affiliations:** 1 International Center for Young Scientists (ICYS), National Institute for Materials Science (NIMS), 1-1 Namiki, Tsukuba, Ibaraki 305-0044, Japan; 2 Center for Functional Sensor & Actuator (CFSN), National Institute for Materials Science (NIMS), 1-1 Namiki, Tsukuba, Ibaraki 305-0044, Japan; YOSHIKAWA.Genki@nims.go.jp; 3 International Research Center for Materials Nanoarchitectonics (MANA), National Institute for Materials Science (NIMS), 1-1 Namiki, Tsukuba, Ibaraki 305-0044, Japan; 4 Materials Science and Engineering, Graduate School of Pure and Applied Science, University of Tsukuba, 1-1-1 Tennodai, Tsukuba, Ibaraki 305-8571, Japan

**Keywords:** Membrane-type Surface stress Sensor (MSS), nanomechanical sensors, static mode operation, interface, finite element analysis (FEA)

## Abstract

Nanomechanical sensors and their arrays have been attracting significant attention for detecting, discriminating and identifying target analytes. The sensing responses can be partially explained by the physical properties of the receptor layers coated on the sensing elements. Analytical solutions of nanomechanical sensing are available for a simple cantilever model including the physical parameters of both a cantilever and a receptor layer. These analytical solutions generally rely on the simple structures, such that the sensing element and the receptor layer are fully attached at their boundary. However, an actual interface in a real system is not always fully attached because of inhomogeneous coatings with low affinity to the sensor surface or partial detachments caused by the exposure to some analytes, especially with high concentration. Here, we study the effects of such macroscopic interfacial structures, including partial attachments/detachments, for static nanomechanical sensing, focusing on a Membrane-type Surface stress Sensor (MSS), through finite element analysis (FEA). We simulate various macroscopic interfacial structures by changing the sizes, numbers and positions of the attachments as well as the elastic properties of receptor layers (e.g., Young’s modulus and Poisson’s ratio) and evaluate the effects on the sensitivity. It is found that specific interfacial structures lead to efficient sensing responses, providing a guideline for designing the coating films as well as optimizing the interfacial structures for higher sensitivity including surface modification of the substrate.

## 1. Introduction

Nanomechanical sensors and their arrays have gained significant attention as a powerful tool for detecting, discriminating and identifying target analytes [1,2,3,4], especially various odors composed of a complex mixture of gaseous molecules [5,6,7]. The versatility of these sensors and their arrays are based on physical and chemical properties of a receptor layer coated on a sensing element. In the case of so-called static mode operation, sensing signals are given by mechanical stress/strain induced by sorption of target molecules in a receptor layer. To obtain high sensitivity in nanomechanical sensing, it is important to efficiently transduce the mechanical stress/strain derived from the deformation of the receptor layers to the sensing elements. In the practical conditions, however, coating films and the surface of sensing elements frequently have different affinities, reflecting the chemical properties of each material, such as organic polymers and an inorganic silicon substrate, leading to poor attachments at their interface and a reduction in the efficiency of the mechanical transduction.

For theoretical investigation of the nanomechanical sensing, there are several analytical solutions, especially for a simple cantilever model. For example, the displacement of a free end of a cantilever (Δ*z*) induced by isotropic internal strain in a receptor layer (ε*_f_*) is given by the following equation [8]:

(1) $$\Delta z=\frac{3l^{2}\left(t_{f}+t_{s}\right)}{\left(A+4\right)t_{f}^{2}+\left(A^{-1}+4\right)t_{s}^{2}+6t_{f}t_{s}}\varepsilon_{f},$$

with:

(2) $$A=\frac{E_{f}w_{f}t_{f}}{1-\nu_{f}}/\frac{E_{s}w_{s}t_{s}}{1-\nu_{s}},$$

where the subscripts “*f* ” and “*s*” denote the coating film and the cantilever substrate, respectively, and *l*, *t*, *w*, *E*, and *ν* correspond to length, thickness, width, Young’s modulus and Poisson’s ratio, respectively. The internal strain in a receptor layer (ε*_f_*) can be replaced by other parameters, such as three-dimensional internal stress in the coating film (*σ_f_*; in the unit of [N m^−2^]) or two dimensional surface stress (*σ*_surf._; in the unit of [N m^−1^]), via the relations $\varepsilon_{f}=\sigma_{f}\left(1-\nu_{f}\right)/E_{f}$ or $\sigma_{f}=\sigma_{\mathrm{surf}.}/t_{f}$ [8,9,10]. When a cantilever is covered with a thin film having a same width (*t_s_* >> *t_f_* and *w_s_* = *w_f_*), Equation (1) reduces to the following equation, which is known as the Stoney’s equation [9]:

(3) $$\Delta z=\frac{3l^{2}\left(1-\nu_{s}\right)}{E_{s}t_{s}^{2}}\sigma_{\mathrm{surf}.}.$$

However, these models are limited to simple analytical problems, and it is still difficult to expand these models to complex problems, especially to the systems having various interfacial structures. On the other hand, it is possible to simulate numerical solutions by using finite element analysis (FEA) even for such complex problems of the nanomechanical sensors [3,8,11,12,13,14,15,16]. 

In the present study, we investigate sensing responses of nanomechanical sensors, focusing on the interfacial structures between a coating film and a substrate of the sensors. Since the available analytical solutions of nanomechanical sensing so far are based on the model in which the coating layer is fully attached on the surface of the sensing element, it is impossible to apply the solutions to complicated interfacial structures, such as partially attached models at their boundary. Thus, we perform numerical calculations of the various interfacial attachment models through FEA using COMSOL Multiphysics^®^ 5.4 with the Structural Mechanics module. We focus on a nanomechanical sensor, especially a Membrane-type Surface stress Sensors (MSS), which is one of the optimized nanomechanical sensors for static operation based on the integrated piezoresistive read-out with high sensitivity [3,4,5,6,7,11,12,17,18,19,20].

It should be noted here that we will discuss a macroscopic model in this study, assuming the “ideal interface” at the attached parts as described in Section 2. Accordingly, the microscopic interface phenomena, such as the lap shear or the interfacial slip, reported as the molecular level effects or the finite size effects for surface stress-based signal responses [21,22,23] are not taken into account. The reason for this assumption is based on the experimental observation; we sometimes encounter a sudden decrease in sensing signals when a receptor layer-coated MSS is exposed to some target analytes, indicating a partial detachment of the receptor layer. This kind of partial detachment could be also confirmed by optical microscope or scanning electron microscope observation. Even in the case with such a partially detached receptor layer, we still observe sensing signals and sometimes the signals are even enhanced. Thus, we assume that the sensing signals are significantly affected by the interfacial structures. To understand the effects of these partial interfacial attachments on the nanomechanical sensing including the domain sizes of the partial attachments as well as the distributions of the partial attachment points, we modeled various macroscopic structures at the interface between the membrane surface of MSS and the receptor layers.

## 2. Simulation

**Figure 1:**
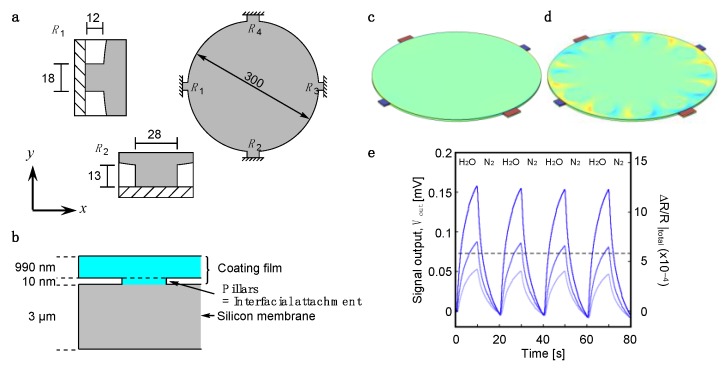
Geometry of a partially attached interface model. (**a**) Configuration of an MSS. The piezoresistor-integrated sensing beams are magnified in the insets. All images are illustrated in top-view. Numbers indicate the dimensions in µm. (**b**) Cross-sectional image of a pillar as an interfacial attachment. The height of pillars is fixed at 10 nm with various radius, *r*_pillar_. (**c,d**) The results of the FEA simulations for the conventional fully attached model (**c**) and partially attached model (**d**), respectively. The distributions of relative resistance changes are plotted as a color gradient. (**e**) Typical signal output of the MSS measured in the experiments. Poly(methyl methacrylate) (PMMA) was coated on the surface of the membrane. *E_f_*, ν*_f_* and *t_f_* are approximately 3 GPa, 0.4 and 1 µm, respectively. Signal outputs were measured at the bias voltage *V_B_* of –1.0 V. The concentrations of water vapor *P_a_/P_o_* are set to 2%, 3% and 5%, where *P_a_* and *P_o_* stand for the partial pressure and saturated vapor pressure, respectively. Black dashed line indicates the level of total resistance change (Δ*R*/*R*|_total_ = 5.82 × 10^−4^) simulated by FEA for the fully attached model (*E_f_* = 3 [GPa], ν*_f_* = 0.4, *t_f_* = 1 [µm], ε*_f_* = 1 × 10^−5^).

To examine the effects of the interfacial structures, pillar-like structures with a fixed height *h* (= 10 [nm]) and varying radius *r*_pillar_ from 1 to 20 µm with the same materials of a coating film are placed between the coating film and the surface of the MSS as the attached points at the interface (Figure 1). Note that these attached points are modeled as “ideal attachments” without assuming any lap shear or interfacial slip. The dimensions for the MSS were set according to the previous report (Figure 1a) [11]. The diameter and the thickness of the membrane were 300 µm and 3 µm, respectively. The membrane is suspended by the four sensing beams, in which piezoresistors are embedded (*R*_1_–*R*_4_). The dimensions of each beam in the directions *x* and *y* are as follows: sensing beams for *R*_1_ and *R*_3_, 12 µm × 18 µm; sensing beams for *R*_2_ and *R*_4_, 28 µm × 13 µm. A receptor layer was set with radius *r_f_* = 150 µm and thickness *t_f_* = 990 nm applying isotropic internal strain, ε*_f_* = 1.0 × 10^−5^. Each geometry was meshed over 20,000 elements, which give sufficient resolution for the present simulation. In the case of an MSS, the surface stress on the membrane is transduced to the four sensing beams as an amplified uniaxial stress, resulting in the changes in electrical resistance of the piezoresistors embedded in the beams. In contrast to simple cantilever-type nanomechanical sensors, in which a displacement of free end Δ*z* (see also Figure S1 in the Supplementary Material) corresponds to a sensing signal, we calculated the total output resistance change Δ*R*/*R*|_total_ obtained from the Wheatstone bridge circuit composed of the four piezoresistors, providing the sensing signals of MSS. The p-type piezoresistors of the MSS are fabricated by doping boron onto a single crystal Si with (100) surface to take advantage of its high piezocoefficient [3,11,24,25,26]. Assuming in-plane stress (i.e., σ*_z_* = 0), relative resistance change can be described as [26,27]:

(4) $$\frac{\Delta R_{i}}{R_{i}}\approx \frac{1}{2}\pi_{44}\left(\sigma_{x}-\sigma_{y}\right),$$

where *π*_44_ (~138 × 10^−11^ [Pa^−1^]) is one of the fundamental piezoresistance coefficients of the silicon crystal, and *σ_x_*, *σ_y_* and *σ_z_* are stresses induced on the piezoresistors in [110], [1–10] and [001] directions of the silicon crystal, respectively. The subscript of “*i*” indicates the position of the piezoresistors on the MSS as illustrated in Figure 1a. The Δ*R*/*R*|_total_ of all four resistors can be approximately given by the following equation:

(5) $${\frac{\Delta R}{R}|}_{\mathrm{total}}=\left(\frac{\Delta R_{1}}{R_{1}}-\frac{\Delta R_{2}}{R_{2}}+\frac{\Delta R_{3}}{R_{3}}-\frac{\Delta R_{4}}{R_{4}}\right),$$

where Δ*R_i_*/*R_i_* is the relative resistance change in *R_i_* (*i* = 1–4) [3,11]. Due to the symmetric geometry, Equation (5) can be reduced to the following equation:

(6) $${\frac{\Delta R}{R}|}_{\mathrm{total}}=2\left(\frac{\Delta R_{1}}{R_{1}}-\frac{\Delta R_{2}}{R_{2}}\right).$$

The signal output of the full Wheatstone bridge (*V*_out_) is given by:

(7) $${V_{\mathrm{out}}=\frac{V_{B}}{4}\frac{\Delta R}{R}|}_{\mathrm{total}},$$

where *V_B_* is bias voltage applied to the bridge [3,11]. Typical signal responses of poly(methyl methacrylate) (PMMA) for water vapors are shown in Figure 1e (see also the Supplementary Material for detailed sensing measurement). A fixed constraint was applied on the outer edges of the sensing beams (Figure 1a).

## 3. Results and Discussion

**Figure 2:**
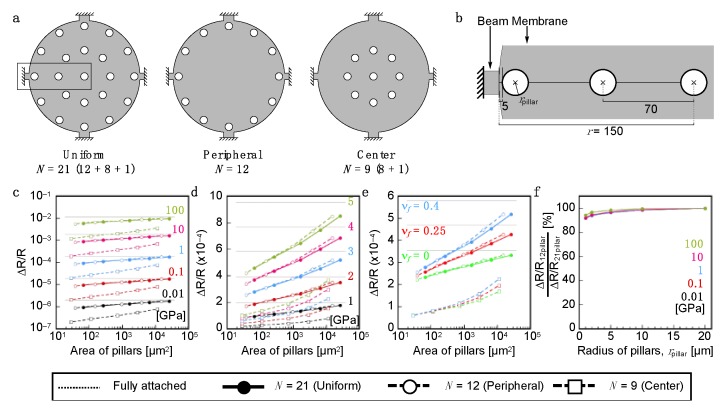
Dependence of the relative resistance change Δ*R*/*R*|_total_ on the area of the interfacial attachments calculated by FEA. (**a**) Configuration of pillars as a model of interfacial attachment. (*Left*) Uniformly distributed model, number of pillars, *N* = 21; (*center*) Peripheral position model, *N* = 12; and (*right*) Center-distributed model, *N* = 9. (**b**) Magnified configuration of pillars shown in the rectangular in (**a**). Numbers indicate the dimensions in µm. The radii of pillars (*r*_pillar_) are varied from 1 µm to 20 µm. (**c,d**) Dependence of the total resistance change (Δ*R*/*R*|_total_) on Young’s moduli as a function of the area of pillars. The Young’s moduli (*E_f_*) are varied from 0.01 GPa to100 GPa (**c**) and from 1 GPa to 5 GPa (**d**). Poisson’s ratio of the coating film (ν*_f_*) is set at 0.4 and *r*_pillar_ is varied as follows: 1, 2, 5, 10, 20 µm. (**e**) Dependence of the total resistance change (Δ*R*/*R*|_total_) on Poisson’s ratios as a function of the area of pillars. The Young’s modulus of coating film (*E_f_*) is set at 3.0 GPa, which is similar to that of PMMA. All dotted lines in (**c**), (**d**) and (**e**) are Δ*R*/*R*|_total_ of a conventional full attachment model without any pillars. (**f**) The comparison between the uniformly distributed model (*N* = 21) and the peripheral position model (*N* = 12).

To investigate the effects of the domain sizes of the interfacial attached points, we first simulated the interfacial structures with uniformly distributed pillars as a function of area. As presented in Figure 2a,b, 21 pillars were placed on the surface of the membrane of MSS as a model of the attached points. The changes in the relative resistance Δ*R*/*R*|_total_ obtained by Young’s modulus *E_f_* and Poisson’s ratio ν*_f_* of a coating layer are calculated by FEA. To confirm the effects of the area of the interfacial attachments, the radii of the pillars *r*_pillar_ are varied from 1 µm to 20 µm. The detailed parameters are listed in Table S1 in the Supplementary Material. As expected, the larger area of interfacial attachments gives the higher Δ*R*/*R*|_total_ in the wide range of the Young’s moduli of the coating films (Figure 2c,d, solid line with closed circles; see also Figure S2, in the Supplementary Material). As presented in Figure 2e, the Poisson’s ratio of the coating films considerably affected Δ*R*/*R*|_total_; the higher Poisson’s ratio gives higher Δ*R*/*R*|_total_ (see also Figure S3, in the Supplementary Material).

**Figure 3:**
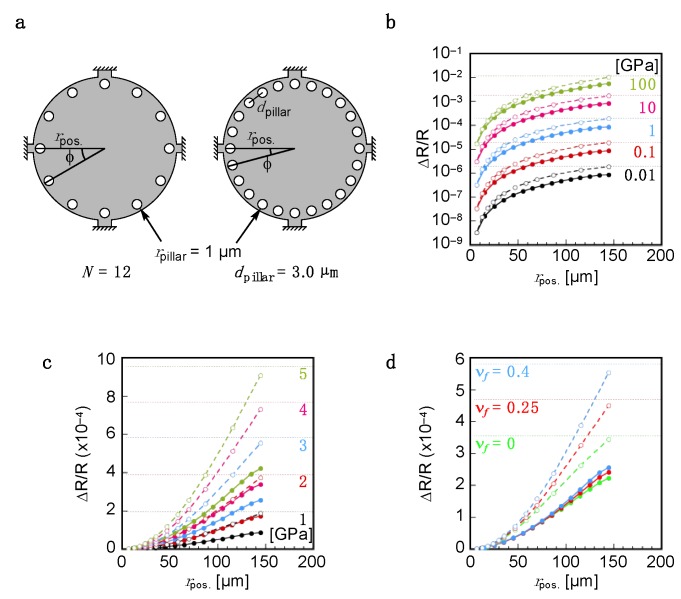
Dependence of the relative resistance change (Δ*R*/*R*|_total_) on the position of the interfacial attachments (*r*_pos._) calculated by FEA. (**a**) Configuration of pillars as a model of interfacial attachment. The radius of pillars (*r*_pillar_) is fixed at 1 µm. (*Left*) Number-fixed model. The number of the pillars (*N*) is fixed at 12. (*Right*) Distance-fixed model. Center-to-center distance between pillars is fixed at ca. 3 µm, indicating that the shortest distance between pillars is 1 µm. Detailed distances are listed in Table 1 and Table 2. (**b**–**c**) Dependence of the total resistance change Δ*R*/*R*|_total_ on Young’s modulus as a function of the position of pillars (*r*_pos._). The Young’s moduli (*E_f_*) are varied from 0.01 GPa to 100 GPa (**b**) and from 1 GPa to 5 GPa (**c**). Poisson’s ratio of a coating film (ν*_f_*) is fixed at 0.4. (**d**) Dependence of the total resistance change Δ*R*/*R*|_total_ on Poisson’s ratio as a function of the position of pillars (*r*_pos._). The Young’s modulus of coating film is fixed at 3.0 GPa, which is similar to that of PMMA. Dotted lines in (**b**), (**c**) and (**d**) are Δ*R*/*R*|_total_ of the full attachment model.

To investigate the effects of the distributions of the pillars, we then investigate the position dependence of pillars. When the pillars were placed at the position close to the center of the membrane surface (the right model in Figure 2a, *N* = 9), the changes in the relative resistance Δ*R*/*R*|_total_ significantly decreased (Figure 2c,d, dashed lines with open squares). On the other hand, interestingly, 12 pillars placed only at the peripheral position exhibited similar level of Δ*R*/*R*|_total_ (Figure 2a,c,d, dashed lines with open circles), especially for the pillars with larger area (*r*_pillar_ ≥ 10 µm) (Figure 2f). To confirm this position dependence further, we simulated two different models as illustrated in Figure 3a: (*i*) pillars placed as a function of position, *r*_pos._, with a fixed number of pillars (Number-fixed model, *N* = 12), and (*ii*) pillars placed as a function of position, *r*_pos._, with a fixed distance between the center to center of pillars (Distance-fixed model, *d*_pillar_ = 3 µm). We fixed the radius of pillars, *r*_pillar_, at 1 µm in both models. Detailed positions and their parameters are listed in Table 1 and Table 2. As observed in the result of FEA as a function of position, *r*_pos._, the positions strongly affected Δ*R*/*R*|_total_ in both Number-fixed and Distance-fixed models (Figure 3b–d; see also Supplementary Material for detailed data). Comparing the models with pillars placed near the center and the peripheral positions, Δ*R*/*R*|_total_ values of the latter cases reached approximately 300 times and 600 times higher than those of the former cases in the cases of Number-fixed and Distance-fixed models (*r*_pos._ = 6.8 µm), respectively. The obtained values are in good agreement with the conventional fully attached models, which have a receptor layer with a radius *r_f_* = *r*_pos._ (Figures S4 and S5 in the Supplementary Material). These results indicate that *r*_pos._ significantly affects the effective coverage of each receptor layer, sometimes resulting in the significant loss of the deformation-induced surface stress applied to the membrane surface of MSS.

**Figure 4:**
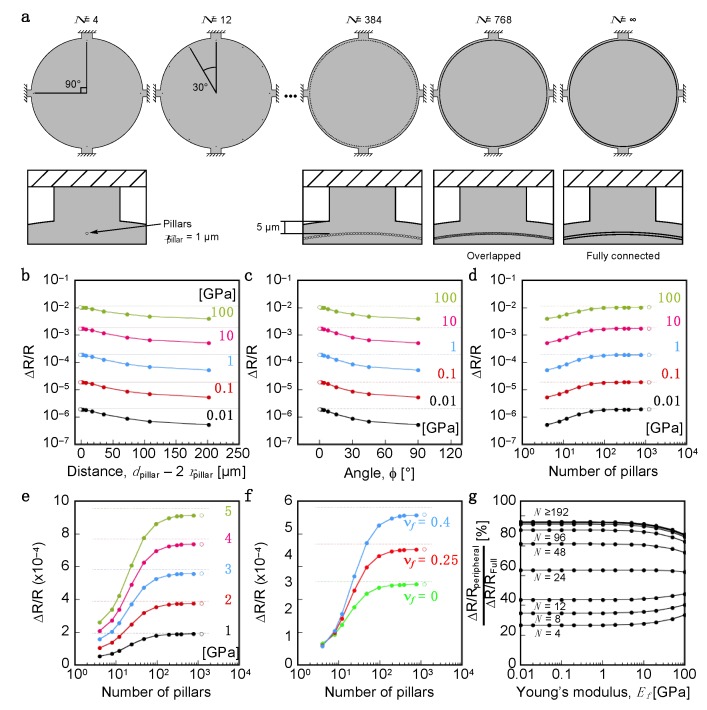
Dependence of the relative resistance change (Δ*R*/*R*|_total_) on the distance of the interfacial attachments (*d*_pillar_) calculated by FEA. (**a**) Configuration of pillars as a model of interfacial attachment. The radius of pillars (*r*_pillar_) is fixed at 1 µm. Each piezoresistor-integrated sensing beam is magnified in the bottom insets. (**b**–**d**) The Young’s modulus-dependent total resistance change Δ*R*/*R*|_total_ on the distance (**b**), area (**c**) and number (**d**) of the pillars. Details are listed in Table 3. The Young’s moduli are varied in the range of 0.01 GPa to 100 GPa. (**e**) Dependence of Δ*R*/*R*|_total_ as a function of the number of pillars. *E_f_* = 1–5 [GPa]. Poisson’s ratio of a coating film (ν*_f_*) is 0.4. (**f**) The Poisson’s ratio-dependent total resistance change (Δ*R*/*R*|_total_) as a function of the position of pillars (*r*_pos._). The Young’s modulus of coating film is 3.0 GPa, which is similar to that of PMMA. Closed and open circles are Δ*R*/*R*|_total_ on *d*_pillar_ and fully attached model, respectively. Dotted lines in (**b**)–(**f**) are Δ*R*/*R*|_total_ of the fully attached model. (**g**) Relative sensing responses compared to Δ*R*/*R*|_total_ of the fully attached model.

It should be noted that pillars placed at the peripheral position in the case of Distance-fixed model (Figure 3) provide higher Δ*R*/*R*|_total_ than that of Number-fixed model with *r*_pillar_ = 20 µm (Figure 2), even though the area of Distance-fixed model with *r*_pillar_ = 1 µm (942 µm^2^; Table 2) is only 6% of Number-fixed model with *r*_pillar_ = 20 µm (15,080 µm^2^; Table S1 in the Supplementary Materials). This result indicates that the distance between interfacial attachments (*d*_pillar_) affects Δ*R*/*R*|_total_ more significantly than the area of interfacial attachments (*r*_pillar_). To confirm this aspect, we constructed the modified model as illustrated in Figure 4a. The pillars were placed at the peripheral position *r*_pos._ = 145 [µm] with the radius of pillars *r*_pillar_ = 1 [µm] by varying the number of pillars ranging from 4 to 768. In addition, we also constructed the fully-connected-pillars model (Figure 4a). Detailed positions and their parameters are listed in Table 3. Figure 4b–d show the effects on Δ*R*/*R*|_total_ as a function of distance (*d*_pillar_), angle (*φ*), and number of pillars (*N*), respectively. It has been found that the higher signal response can be obtained with shorter distances, and Δ*R*/*R*|_total_ with the shortest distance of pillars (*d*_pillar_ − 2 × *r*_pillar_) less than 5 µm reached the similar level of the signal response to the fully-connected-pillars model (Figure 4d–g). Notably, the pillars placed nearby the four sensing beams of the MSS yield largest signal response, for example, in the case of the number of pillars *N* = 4, and the distance of the pillars from the sensing beams significantly affects the signal response because of the geometry of the MSS (see also Appendix A).

## 4. Conclusions

In summary, we have demonstrated the FEA simulations for investigating the effects of interfacial structures on the signal responses of nanomechanical sensors, especially MSS. Despite the various advantages of the nanomechanical sensors, it is often difficult to achieve high sensitivity and selectivity because of the low affinity between a coating film and a substrate of the sensing element, including the mechanical detachments between them. To properly investigate the actual contribution of a coating film to a sensing signal, it is important to understand the effects of interfacial attachments on the sensing responses. In the present study, we have shown the effects of the interfacial structures using the several models analyzed by FEA simulations. The effects of the physical parameters of coating films, such as Young’s modulus and Poisson’s ratio are also discussed. It was demonstrated that the attachments at the peripheral positions give the signal responses as high as uniformly distributed or fully attached models, while the attachments at the inner positions of the membrane cannot efficiently transduce the mechanical response of a coating film to the membrane, leading to significant losses of sensing signals. The presented study will provide a strategy for designing the coating films as well as optimizing the interfacial structures for higher sensitivity including surface modification of a substrate [2,28].

## Figures and Tables

**Table 1:** Detailed parameters of the number-fixed model.

Number-Fixed Model, *N* = 12				
*r*_pos._ [µm] *^a^*	*φ* [°] *^a^*	*d*_pillar_ [µm] *^a,b^*	No. of Pillars	Area of Pillars [µm^2^]
145	30	74.5 (72.5)	12	38
135	30	69.4 (67.4)	12	38
125	30	64.2 (62.2)	12	38
115	30	59.0 (57.0)	12	38
105	30	53.8 (53.8)	12	38
95	30	48.7 (46.7)	12	38
85	30	43.5 (41.5)	12	38
75	30	38.3 (36.3)	12	38
65	30	33.1 (31.1)	12	38
55	30	28.0 (26.0)	12	38
45	30	22.8 (20.8)	12	38
35	30	17.6 (15.6)	12	38
25	30	12.4 (10.4)	12	38
15	30	7.25 (5.25)	12	38
6.8	30	3.00 (1.00)	12	38

*^a^ r*_pos._, *φ*, and *d*_pillar_ are denoted in Figure 3a. *^b^* Values in parentheses are the shortest distance between the neighboring pillars (*d*_pillar_ − 2 × *r*_pillar_).

**Table 2:** Detailed parameters of the distance-fixed model.

Distance-Fixed Model, *d*_pillar_ = 3 µm				
*r*_pos._ [µm] *^a^*	*φ* [°] *^a^*	*d*_pillar_ [µm] *^a,b^*	No. of pillars	Area of pillars [µm^2^]
145	1.2	3.02 (1.02)	300	942
115.7	1.5	3.00 (1.00)	240	754
87	2	3.00 (1.00)	180	565
69.8	2.5	3.00 (1.00)	144	452
58.3	3	3.00 (1.00)	120	377
46.8	3.75	3.00 (1.00)	96	302
35.4	5	3.00 (1.00)	72	226
29.7	6	3.00 (1.00)	60	188
23.9	7.5	3.00 (1.00)	48	151
18.2	10	3.00 (1.00)	36	113
12.5	15	3.00 (1.00)	24	75
6.8	30	3.00 (1.00)	12	38

*^a^ r*_pos._, *φ*, and *d*_pillar_ are denoted in Figure 3a. *^b^* Values in parentheses are the shortest distance between the neighboring pillars (d_pillar_ − 2 × r_pillar_).

**Table 3:** Detailed parameters of the model in Figure 4.

No. of Pillars	Angle, *φ* [°]*^a^*	Distance, *d*_pillar_ [µm] *^a,b^*
4	90	204 (202)
12	30	74.5 (72.5)
24	15	37.6 (35.6)
48	7.5	18.8 (16.8)
96	3.8	9.42 (7.42)
192	1.9	4.71 (2.71)
300	1.2	3.02 (1.02)
384	0.94	2.36 (0.36)
768	0.47	1.18 (−0.82)

*^a^ φ* and *d*_pillar_ are denoted in Figure 3a; *^b^* Values in parentheses are the shortest distance between the neighboring pillars (*d*_pillar_ − 2 × *r*_pillar_).

**Figure A1:**
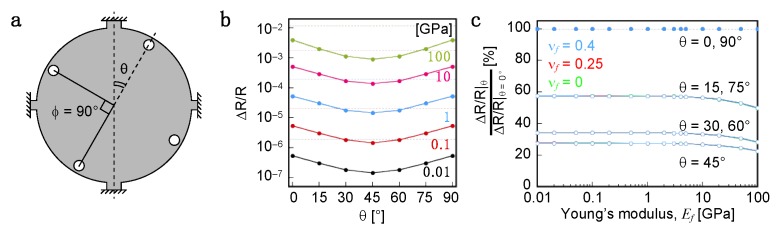
Dependence of the relative resistance change (Δ*R*/*R*|_total_) on the position of the interfacial attachments from the four sensing beams of the MSS calculated by FEA. (**a**) Configuration of pillars as a model of interfacial attachment. The radius of pillars (*r*_pillar_) and the number of pillars (*N*) are fixed at 1 µm and 4, respectively, with the angle *φ* = 90°. (**b**) Dependence of the total resistance change Δ*R*/*R*|_total_ on Young’s modulus as a function of the angle of pillars from *y* axis. The Young’s moduli (*E_f_*) are varied from 0.01 GPa to 100 GPa. Poisson’s ratio of a coating film (ν*_f_*) is fixed at 0.4. Dotted lines are Δ*R*/*R*|_total_ of the fully attached models. (**c**) Dependence of the relative resistance change Δ*R*/*R*|_total_ on Young’s modulus and Poisson’s ratio.

## Supplementary Materials

Supplementary Materials: The following are available online at https://www.mdpi.com/1424-8220/20/5/1518/s1, Supplementary text: Detailed experimental procedure, Figure S1: Distribution of displacements and relative resistance change on the interfacial structure, Figures S2, S3, and S5: the distributions of relative resistance changes, Figure S4: Comparison between the distance-fixed model and fully attached model with different radius of a receptor layer, Table S1; The list of detailed parameters of the interfacial structures.

## Appendix A

Owing to the geometry of the MSS, the distance between pillars and the four sensing beams significantly affects the signal response. As illustrated in Figure A1a, four pillars are placed at the peripheral position (*r*_pos_ = 145) with varied angle *θ* [°] from the *y* axis (0° ≤ *θ* < 90°). As presented in Figure A1b,c, the relative resistance change (Δ*R*/*R*|_total_) dramatically decreases and reaches ca. 30% at *θ* = 45° compared to Δ*R*/*R*|_total_ (*θ* = 0°). While the Young’s modulus of the coating films (*E_f_*) are slightly affects the signal responses, the Poisson’s ratio of the coating films (ν*_f_*) has almost effects on Δ*R*/*R*|_total_ (Figure A1c)
