# Supplementary material for: Finite Element Analysis of Interface Dependence on Nanomechanical Sensing
Source: Sensors (Basel). 2020 Mar 10;20(5):1518. doi: 10.3390/s20051518 (PMC7085745; doi:10.3390/s20051518)
Supplement: Supplementary file 1 [file sensors-20-01518-s001.pdf]

Supplementary materials

Finite element analysis of interface dependence on nanomechanical sensing

Kosuke Minami [1,2,*] and Genki Yoshikawa [2,3,4]

[1] International Center for Young Scientists (ICYS), National Institute for Materials Science (NIMS), 1-1 Namiki, Tsukuba, Ibaraki 305-0044, Japan

[2] Center for Functional Sensor & Actuator (CFSN), National Institute for Materials Science (NIMS), 1-1 Namiki, Tsukuba, Ibaraki 305-0044, Japan; YOSHIKAWA.Genki@nims.go.jp

[3] International Center for Materials Nanoarchitectonics (MANA), National Institute for Materials Science (NIMS), 1-1 Namiki, Tsukuba, Ibaraki 305-0044, Japan

[4] Materials Science and Engineering, Graduate School of Pure and Applied Science, University of Tsukuba, 1-1-1 Tennodai, Tsukuba, Ibaraki 305-8571, Japan.

* Correspondence: MINAMI.Kosuke@nims.go.jp

1. Supplementary Methods

Materials. Poly(methyl methacrylate) (PMMA; Mw ~ 15000) and *N,N*-dimethylformamide (DMF) was purchased from Sigma-Aldrich Inc. and used as received. Ultrapure water purified by Millipore (Tokyo, Japan) was used for water vapor.

Fabrication of an MSS receptor layer. Poly(methyl methacrylate) (PMMA) were dissolved in DMF at the concentration of 1 mg mL^{-1}. The solution of PMMA was deposited on a channel of the MSS chip with an inkjet spotter (LaboJet-500SP, MICROJET Corporation) equipped with a nozzle (IJHBS-300, MICROJET Corporation).

Sensing system and procedure. We performed the sensing experiments as reported in our previous paper [1]. The MSS chip coated with PMMA was placed in a Teflon chamber and placed in an incubator at a controlled temperature of 25.0 ± 0.1 °C. The chamber was connected to a gas system consisting of two mass flow controllers (MFCs), a mixing chamber, a purging gas line and a vial for a target sample liquid (in this case, water) in an incubator at a controlled temperature of 15.0 ± 0.1 °C. The vapors of water were produced by bubbling with a carrier gas. Pure nitrogen gas was used as carrier and purging gases. The total flow rate was maintained at 100 mL min^{-1} during the experiments. The concentration of vapors was controlled by MFC-1 with P_a/P_o at 2, 3 and 5%, where P_a and P_o stand for a partial vapor pressure and the saturated vapor pressure of water, respectively.

Before measuring MSS signals, pure nitrogen gas was introduced into the MSS chamber for 1 min; subsequently the MFC-1 (sampling line) and MFC-2 (purging line) were switched every 10 s with a controlled total flow rate of 100 mL min^{-1} for 4 cycles (Figure 1e). Sensing signals were measured at a bias voltage V_B of –1.0 V applied to the bridge circuit, and the relative resistance changes of piezoresistors with a sampling rate of 20 Hz were recorded. The measurement system program was designed by LabVIEW (National Instruments Corporation).

2. Supplementary Figures

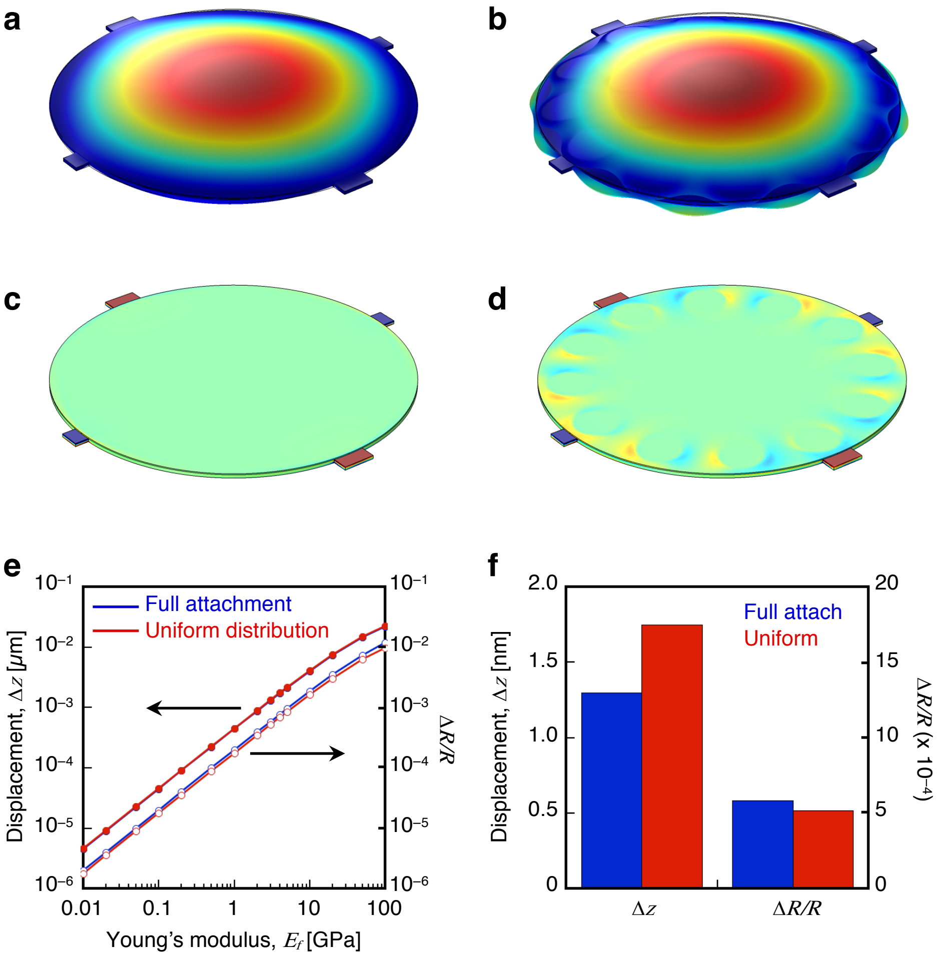

Figure S1. Distribution of displacements and relative resistance change on the interfacial structure. Results of the FEA simulations of conventional fully attached model (**a,c**) and uniformly distributed model (**b,d**), respectively. (**a,b**) The distributions of displacements in the normal direction (z). (**c,d**) The distributions of the relative resistance changes. The Young's modulus and Poisson's ratio of each receptor layer shown in this figure are 3 GPa and 0.4, respectively. (**e,f**) Comparison between the displacements (Δz) and relative resistance change ($\Delta R/R$).

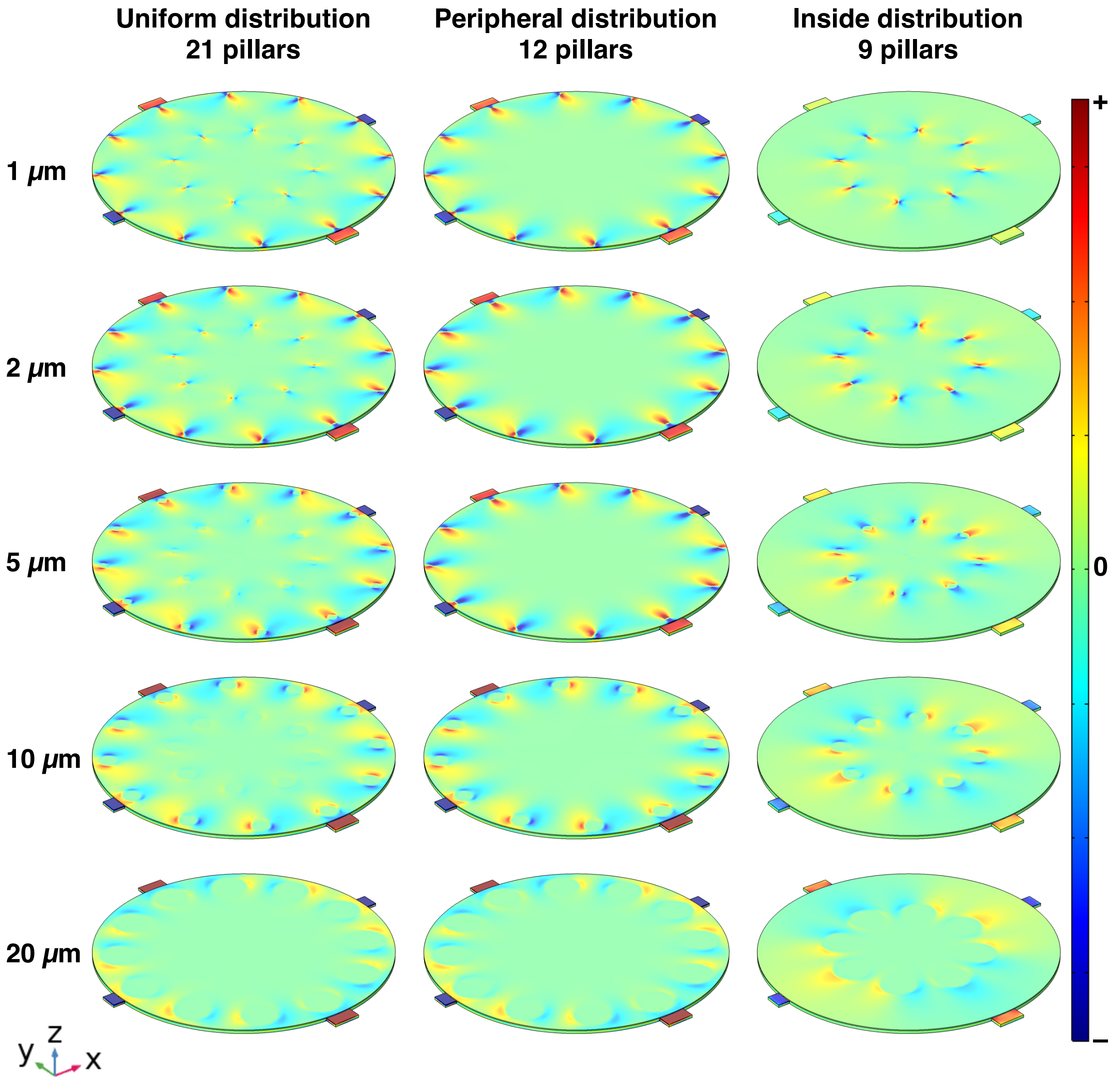

Figure S2. Distributions of relative resistance changes on the radius of pillars. Results of the FEA simulations for uniform (*Left*), peripheral (*Center*) and inside distribution models (*Right*), respectively, with varying the radius of pillars (r_{pillar}) from 1 μm to 20 μm. The distributions of relative resistance changes are plotted as a color gradient. The Young's modulus and Poisson's ratio of a coating film shown in this figure are fixed at 3.0 GPa and 0.4, respectively. $\varepsilon_f = 1.0 \times 10^{-5}$.

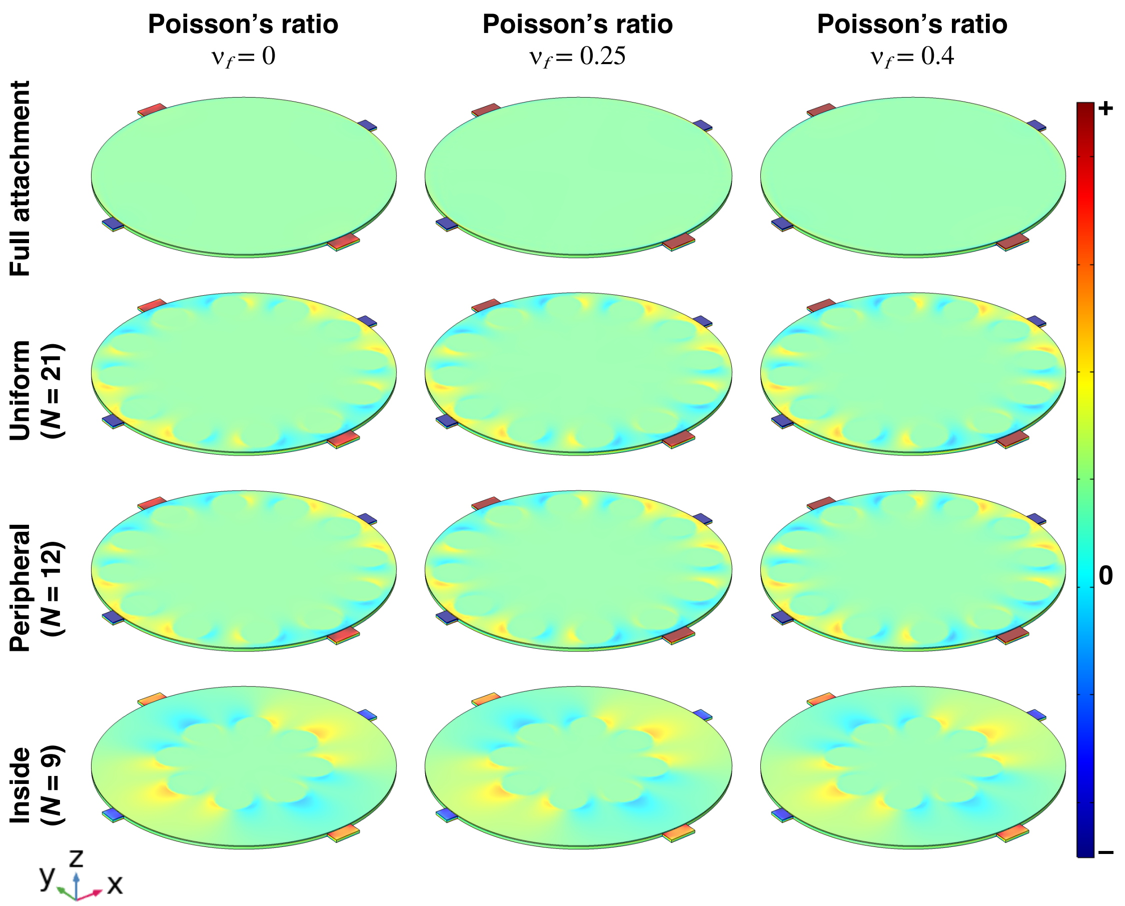

Figure S3. Distributions of relative resistance changes on the Poisson's ratio of the coating film. Results of the FEA simulations for fully attached model and uniform, peripheral and inside distribution models. For the interfacial attachment models, the radius of pillars (r_{pillar}) is fixed at 20 μm. The distributions of relative resistance changes are plotted as a color gradient. The Young's modulus of a coating film shown in this figure is fixed at 3.0 GPa. $\varepsilon_f = 1.0 \times 10^{-5}$.

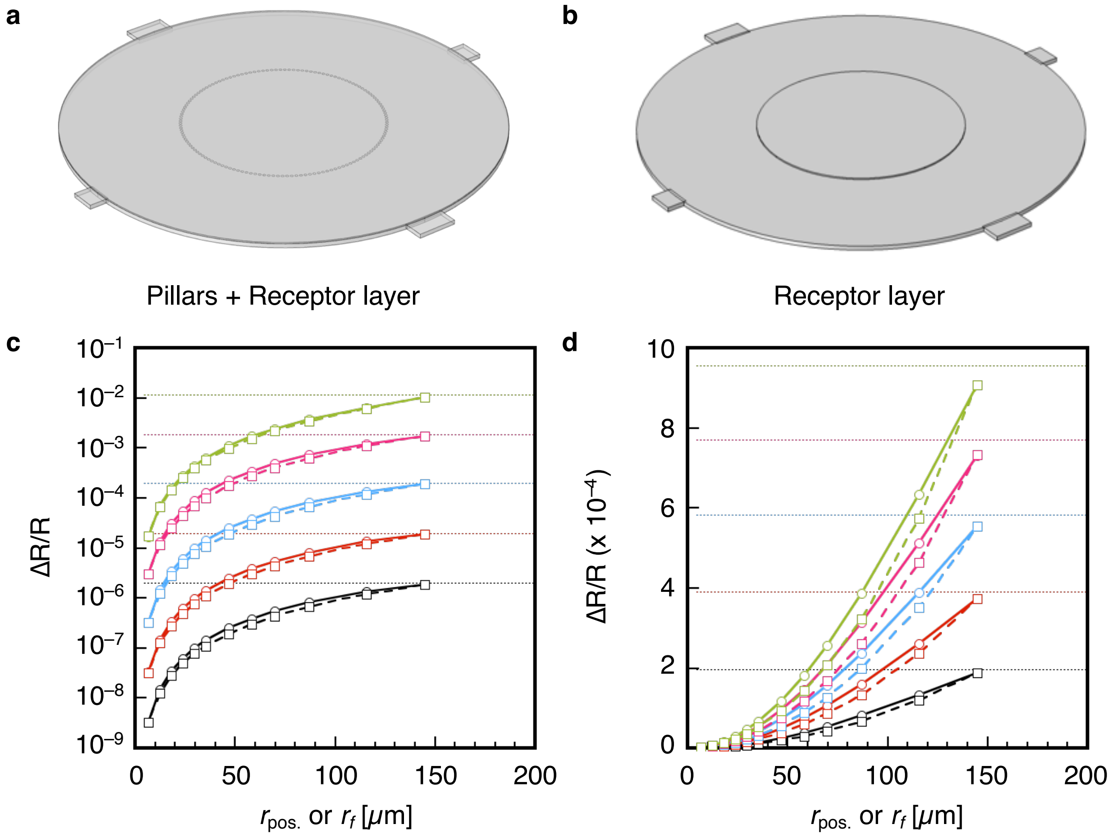

Figure S4. Comparison between the Distance-fixed model and the fully attached model, in which radius of receptor layers (r_f) is fixed at $r_{pos.}$. (a) Configuration of the structure with Distance-fixed model ($r_{pos.}$ = 70 μm). See also Figure 3 in the main text. (b) Configuration of fully attached model with $r_f = r_{pos.}$. (c–d) The Young's modulus-dependent total resistance change $\Delta R/R|_{total}$ as a function of the position of pillars ($r_{pos.}$) or the radius of the coating film (r_f). The Young's moduli (E_f) are varied from 0.01 GPa to 100 GPa (b) and from 1 GPa to 5 GPa (c). Poisson's ratio of a coating film (ν_f) is 0.4. Dotted lines in (c) and (d) are $\Delta R/R|_{total}$ of the fully attached model (r_f = 150 [μm]).

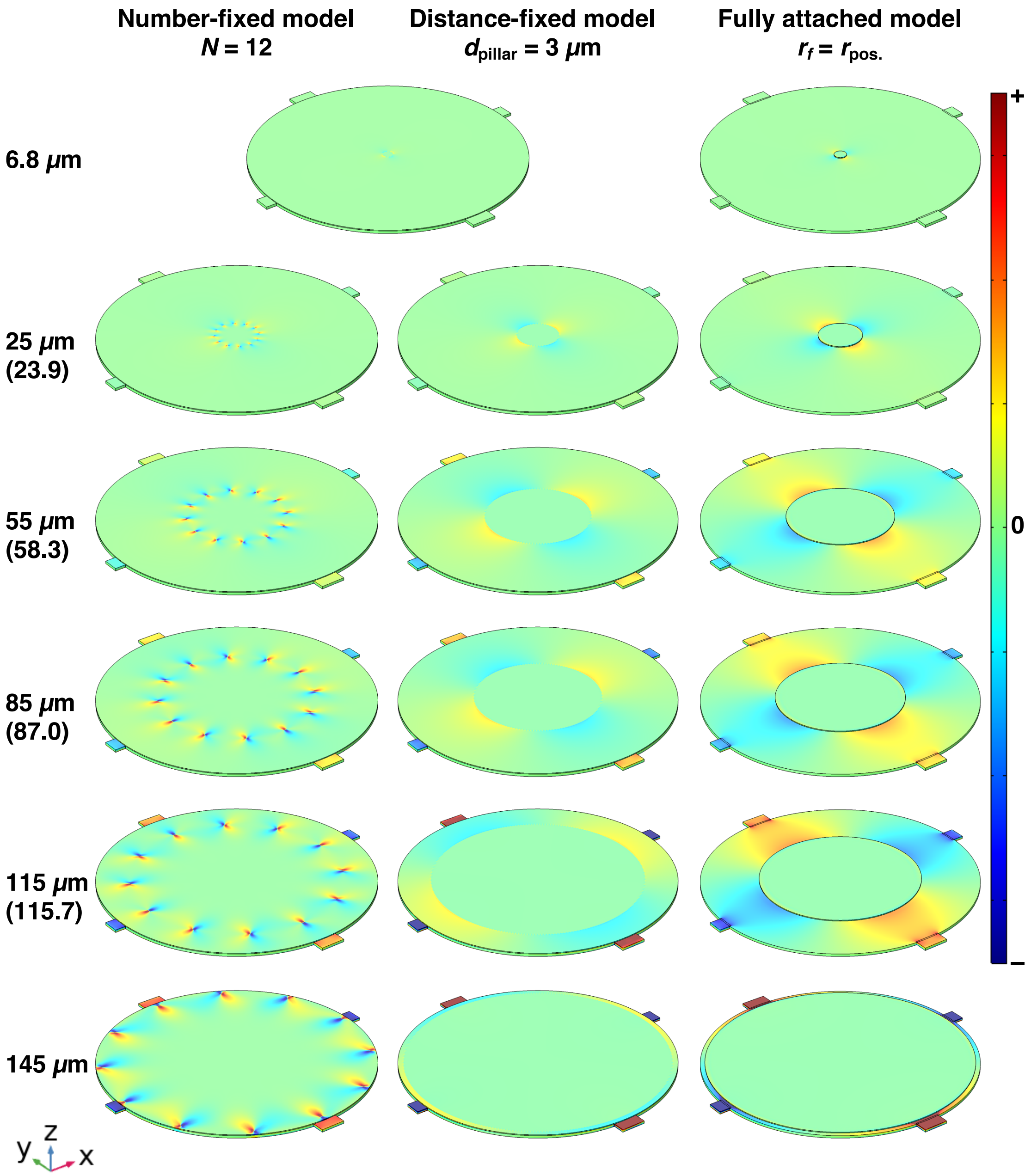

Figure S5. Distribution of relative resistance change. Results of the FEA simulations for the Number-fixed (*Left*), Distance-fixed (*Center*) and fully attached models (*Right*), respectively. The distributions of relative resistance changes are plotted as a color gradient. The Young's modulus and Poisson's ratio of a coating film shown in this figure are fixed at 3.0 GPa and 0.4, respectively. $\varepsilon_f = 1.0 \times 10^{-5}$.

3. Supplementary Tables

Table S1. Detailed parameters of the model of interfacial structures shown in Fig. 2.

	No. of pillars	r_{pillar} [μm][a]	Area of Pillars [μm^2]	**Peripheral** $r_{pos.}$ [μm][b] ($r_{pos.} - r_{pillar}$)[c]	**Peripheral** d_{pillar} [μm][b] ($d_{pillar} - 2 \times r_{pillar}$)[d]	**Middle** $r_{pos.}$ [μm][b] ($r_{pos.} - r_{pillar}$)[c]	**Middle** d_{pillar} [μm][b] ($d_{pillar} - 2 \times r_{pillar}$)[d]	**Center** $r_{pos.}$ [μm][b] ($r_{pos.} - r_{pillar}$)[c]
**Uniform distribution**	21	1	66	145 (144)	74.5 (72.5)	71 (70)	53.6 (51.6)	1 (0)
		2	264	145 (143)	74.0 (70.0)	72 (70)	53.6 (49.6)	2 (0)
		5	1649	145 (140)	72.5 (62.5)	75 (70)	53.6 (43.6)	5 (0)
		10	6597	145 (135)	69.9 (49.9)	80 (70)	53.6 (33.6)	10 (0)
		20	26389	145 (125)	64.7 (24.7)	90 (70)	53.6 (13.6)	20 (0)
**Peripheral distribution**	12	1	28	145 (144)	74.5 (72.5)	–	–	–
		2	113	145 (143)	74.0 (70.0)	–	–	–
		5	707	145 (140)	72.5 (62.5)	–	–	–
		10	2827	145 (135)	69.9 (49.9)	–	–	–
		20	11310	145 (125)	64.7 (24.7)	–	–	–
**Center distribution**	9	1	38	–	–	71 (70)	53.6 (51.6)	1 (0)
		2	151shortest	–	–	72 (70)	53.6 (49.6)	2 (0)
		5	942	–	–	75 (70)	53.6 (43.6)	5 (0)
		10	3770	–	–	80 (70)	53.6 (33.6)	10 (0)
		20	15080	–	–	90 (70)	53.6 (13.6)	20 (0)

[a] r_{pillar} is denoted the radius of pillars as shown in Fig. 2(b) in main text.
[b] $r_{pos.}$ and d_{pillar} are denoted as the position of pillars from center of the membrane and the shortest distance between the centers of neighboring pillars as shown in Fig. 3(a) in main text.
[c] Values in parentheses are the center position of pillars from the center of the membrane ($r_{pos.} - r_{pillar}$).
[d] Values in parentheses are the distance between the neighboring pillars ($d_{pillar} - 2 \times r_{pillar}$).

1. Minami, K.; Shiba, K.; Yoshikawa, G., Discrimination of structurally similar odorous molecules with various concentrations by using a nanomechanical sensor. *Anal. Methods* **2018,** *10*, 3720-3726.
